# Effects of essential oils on egg production and feed efficiency as influenced by laying hen breed: A meta-analysis

**DOI:** 10.14202/vetworld.2024.197-206

**Published:** 2024-01-23

**Authors:** Arif Darmawan, Ergin Öztürk, Emrah Güngör, Şevket Özlü, Anuraga Jayanegara

**Affiliations:** 1 Department of Animal Nutrition and Feed Technology, Faculty of Animal Science, IPB University, Bogor, Indonesia; 2 Department of Animal Science, Faculty of Agriculture, Ondokuz Mayis University, Samsun, Turkey; 3 Animal Feed and Nutrition Modelling Research Group, Animal Science Faculty, IPB University, Bogor, Indonesia

**Keywords:** antioxidant enzyme, egg production, egg quality, gut health, serum biochemistry

## Abstract

**Background and Aim:**

Successful rearing of laying hens to achieve optimal egg production is an endeavor that often faces various constraints and challenges, such as infectious diseases, environmental stressors, and fluctuations in feed quality. The incorporation of essential oils (EOs) into the diet of laying hens has attracted considerable attention in recent years. Therefore, our study aimed to evaluate the efficacy of EO inclusion in laying hen diets by considering the effects of production phase and breed on performance, egg quality, serum biochemistry, gut health, and antioxidant activity.

**Materials and Methods:**

The articles were obtained from the Web of Science, Scopus, Science Direct, and PubMed using the search terms “essential oils,” “laying hens,” and “phytobiotics.” Data from 27 articles and 71 experiments were grouped according to laying hen production phase and breed in the database. The EO levels ranged from 0 to 1000 mg/kg, with thymol and carvacrol being the major EOs. A mixed model was used to analyze the data. Random effects were applied to the treatment, and fixed effects were applied to EO level, production phase, and breed.

**Results:**

Egg production, feed intake, feed efficiency, eggshell quality, villus height, crypt depth, superoxide dismutase, and glutathione peroxidase levels increased linearly (p = 0.05) and egg weight and mass increased quadratically (p < 0.05) with increasing EO concentrations. An interaction was observed between the EO level egg production and feed conversion ratio (p = 0.05). Serum glucose, cholesterol, and malondialdehyde levels decreased with increasing EO concentrations (p < 0.05).

**Conclusions:**

The inclusion of EOs effectively increased egg production, feed efficiency, egg weight, egg mass, eggshell quality, oxidative enzymes, and intestinal health. In addition, the proportion of dietary EOs in lightweight laying hens was higher than that in semi-heavy-weight laying hens in improving egg production and feeding efficiency.

## Introduction

As the global demand for poultry products continues to increase, the main objectives are to optimize egg production and ensure the welfare of laying hens. However, the successful rearing of laying hens to achieve optimal egg production often faces various obstacles and challenges. These problems include health issues such as infectious diseases, environmental stressors, and fluctuations in feed quality. In recent years, there has been an increasing interest in the use of essential oils (EOs) as a potential solution to overcome these constraints and improve laying hen performance. EOs are natural aromatic compounds extracted from plants and have various bioactive properties, including antimicrobial, anti-inflammatory, and antioxidant effects. Terpenoid components, such as menthol, thymol, linalool, carvacrol, geraniol, eugenol, and cinnamaldehyde, have been suggested to be beneficial as antimicrobial, antioxidant, and digestive enzyme stimulants [1]. Carvacrol and thymol are the main components of oregano EOs, which are the strongest terpenoids used as antimicrobials and antioxidants, with beneficial effects on intestinal function and poultry performance [2, 3].

The favorable effects of EOs include enhancement of antioxidant enzymes, such as superoxide dismutase (SOD) and glutathione peroxidase (GSHPx) [4], and lowering of *Escherichia coli* and *Salmonella* populations in the intestines [5], thereby supporting the health and productivity of laying hens. Previous studies have evaluated the positive effects of dietary EO inclusion on egg production, eggshell thickness, and intestinal morphology [6, 7]. However, not all studies have demonstrated the positive effects of dietary EOs. Olgun [8] and Feng *et al*. [9] reported that EO inclusion had no influence on hen-day productivity or feed efficiency, and instead lowered egg production [10]. Inconsistent results have been identified among studies, which could be due to several factors such as plant source, form, method of inclusion, EO level, EO composition, age, and chicken breed [3, 11]. To address this contradiction, several comprehensive reviews on poultry have been conducted [3, 12, 13], including a meta-analysis of EO application in broiler chickens [11]. However, no systematic review has been conducted using a meta-analysis approach that integrates data from various studies on the effects of dietary EOs on laying hens, considering the production phase and breed.

Therefore, our study aimed to evaluate the efficacy of EO inclusion in laying hen diets by considering the effects of the production phase and breed on performance, egg quality, serum biochemistry, gut health, and antioxidant activity.

## Materials and Methods

### Ethical approval

Animal ethics committee approval was not required for this study due to the absence of animal use. This meta-analysis study followed the PRISMA guidelines [14].

### Study period and location

The study was conducted from August 2022 to June 2023 at the Department of Animal Science, Faculty of Agriculture, Ondokuz Mayiz University, Turkey.

### Search strategy

The meta-analysis database was compiled from articles reporting the effects of EOs on laying hen performance, egg quality, serum biochemicals, antioxidant activity, intestinal morphology, and microbial population. The articles were obtained from the Web of Science, Scopus, Science Direct, and PubMed using the search terms “essential oils,” “laying hens,” and “phytobiotics.”

### Selection criteria

The articles selection was conducted with the following criteria: (a) English articles, (b) original article type, (c) open access articles, (d) *in vivo* studies, (e) EO levels were clearly reported, (f) EOs were only added to feed, (g) single EO treatment or no additional interfering treatments, and (h) parameters were clearly reported.

### Inclusion and exclusion criteria

**Figure-1:**
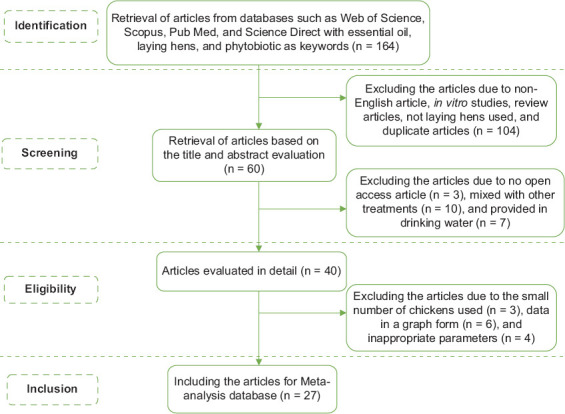
Database article selection process based on the PRISMA method.

We identified 164 articles by title selection. Subsequently, only 27 articles were included in the database (Table-1) [4–10, 15–34] after reviewing the titles, abstracts, and entire contents of the articles in detail. We included studies reporting the effects of feeding EOs on parameters of performance, egg quality, serum biochemicals, microbial population, and gut morphology. In the screening stage, we excluded 104 articles due to non-English articles, in vitro studies, review articles, not laying hens used, and duplicate articles. With more detailed screening, we excluded 20 articles due to no open access article (n = 3), mixed with other treatments (n = 10), and provided in drinking water (n = 7). We selected 40 eligible articles for full content evaluation. We excluded articles due to the small number of chickens used (n = 3), data in a graph form (n = 6), and inappropriate parameters (n = 4). Finally, only 27 articles were included in the meta-analysis database. The article selection process included identification, screening, eligibility, and inclusion criteria based on the PRISMA method (Figure-1). Furthermore, the included articles were classified into several categories, including author name, publication year, EO source, EO component, EO level, age/production phase, and chicken breed. The data from all the parameters were entered into the database by converting them to the same unit. All EO concentrations were converted to milligrams per kg (mg/kg) and ranged from 0 mg/kg (control) to 1000 mg/kg, with thymol and carvacrol as the major EO components. Articles discussing more than one EO source were coded separately. Before further analysis, the data were grouped according to the laying hen production phase (first 50 weeks; second 51 weeks) and laying hen breed: Lightweight (Lohmann LSL-white, Lohmann LSL-Lite, NOVogen White, Hy-Line Leghorn, Lohmann white, Roma, Jing Tint, and Dawu Golden Phoenix) and semi-heavy weight (Hy-Line Brown, Lohmann Brown, ISA brown, and Nick Brown).

**Table-1:** Studies included in the database.

Reference	Source	Main EO compounds	EO levels (mg/kg)	Breeds	Age (weeks)	Production phase
Puvača *et al*. [4]	*M. alternifolia*	Terpinen-4-ol, γ-terpinene	0–80	Lohmann Brown (SHW)	55–58	Second
Mousavi *et al*. [5]	*T. vulgaris, R. officinalis, A. graveolens, M. piperita* L.	Thymol, carvacrol, dill ether, cineol, camphor, α-pinene, menthol	0–200	Hy-Line W36 Leghorn (LW)	41–45, 45–70	First, second
Ghanima *et al*. [6]	*R. officinalis*	Cineol, camphor	0–300	ISA Brown (SHW)	28–44, 45–76	First, second
Arslan *et al*. [7]	Commercial EOs	Eugenol, nerolidol, piperine, thymol, linalool, geranio	0–200	Nick Brown (SHW)	48–60	Second
Olgun [8]	*T. vulgaris, N. sativa, F. vulgare, P. anisum, R. officinalis*	Thymol, p-cymene, α-pinene, anethole, cineole	0–600	Super Nick (SHW)	21–33	First
Feng *et al*. [9]	*O. vulgare*	Carvacrol, thymol	0–400	Hy-line Brown (SHW)	60–74	Second
Torki *et al*. [10]	*L. angustifolia, M. spicata*	Linalool, linalyl acetate, carvone, limonene	0–250	Lohmann LSL-Lite (LW)	47–56	Second
Nasiroleslami and Torki [15]	*F. vulgare*	Chavicol, anethole, phellandrene, geraniol, citronellol, borneol	0–300	Lohmann LSL-Lite (LW)	31–37	First
Bozkurt *et al*. [16]	*Origanum* spp., *L. nobilis, S. triloba, M. communis, F. vulgare*	Carvacrol, thymol, cineole, limonene	0–24	Lohmann LSL-Classic (LW)	36–50	First
Bozkurt *et al*. [17]			0–24	Lohmann LSL White (LW), Lohmann Brown (SHW)	22–36	First
Kaya *et al*. [18]	*T. vulgaris, M. piperita*	Carvacrol, thymol, menthol	0–300	Dawu Golden Phoenix (SHW)	55–63	Second
Arpášová *et al*. [19]	*O. vulgare*	Carvacrol, thymol	0–1000	Hy-Line Brown (SHW)	17–40	First
He *et al*. [20]	*O. vulgare*	Carvacrol, thymol	0–150	Hy-Line brown (SHW)	31–38	First
Ding *et al*. [21]	*Commercial EOs*	Thymol, cinnamaldehyde	0–150	Lohmann Brown (SHW)	54–64	Second
Yu *et al*. [22]	*I. verum Hook.*f	Chavicol, anethole	0–600	Hy-Line brown (SHW)	26–34	First
Cufadar [23]	*R. officinalis*	Cineol, camphor, α-pinene	0–250	NOVOgen White (LW)	24–36	First
Cufadar [24]	*O. vulgare*	Carvacrol, thymol	0–250	Super Nick (SHW)	40–52	First
Migliorini *et al*. [25]	*O. vulgare*	Carvacrol, thymol,	0–200	Hy-Line Brown (SHW)	60–68	Second
Migliorini *t al*. [26]	*O. vulgare*	Carvacrol, thymol	0–200	Hy-Line Brown (SHW)	59–71	Second
Gul *et al*. [27]	*O. syriacum* L.	Carvacrol, thymol	0–600	Lohmann White (LW)	22–45	First
Wang *et al*. [28]	Commercial EOs	Thymol	0–450	Roman (LW)	21–30	First
Garcia *et al*. [29]	*R. officinalis*	Cineol, camphor	0–200	Hy Line Brown (SHW)	30–45	First
Beyzi *et al*. [30]	*T. vulgaris*	Thymol, γ-terpinene	0–300	Lohmann White Leghorn (LW)	25–30	First
Ramirez *et al*. [31]	*L. origanoides*	carvacrol, thymol	0–150	ISA Brown (SHW)	70-80	Second
Ghanem *et al*. [32]	*C. zeylanicum*	Eugenol, β-caryophyllene	0–150	Lohman Brown (SHW)	24–36	First
Gao *et al*. [33]	*O. vulgare*	Carvacrol, thymol	0–320	Jing Tint (LW)	58–62	Second
Xiao *et al*. [34]	Commercial EOs	Carvacrol, thymol	0–300	Dawu Golden Phoenix (SHW)	55–63	Second

EOs=Essential oils; LW=Lightweight; SHW=Semi-heavyweight; first, ≤50 weeks; second, ≥51 weeks. *F. vulgare=Foeniculum vulgare, L. nobilis=Laurus nobilis, S. triloba=Salvia triloba, M. communis=Myrtus communis,* *T. vulgaris=Thymus vulgaris, M. piperita=Mentha piperita, O. vulgare=Origanum vulgare, T. vulgaris=Thymus vulgaris, N. sativa=Nigella sativa, F. vulgare=Foeniculum vulgare, P. anisum=Pimpinella anisum, R. officinalis=Rosmarinus officinalis, I. verum Hook.f=Illicium verum Hook.f, R. officinalis=Rosmarinus officinalis, A. graveolens=Anethum graveolens, M. piperita L.=Mentha piperita L., O. syriacum L.=Origanum syriacum L., T. vulgaris=Thymus vulgaris,* *M. alternifolia=Melaleuca alternifolia, L. angustifolia=Lavandula angustifolia, M. spicata=Mentha spicata,* *L. origanoides=Lippia origanoides, C. zeylanicum=Cinnamomum zeylanicum*

The parameters evaluated were hen-day production, feed intake, egg weight and mass, feed conversion ratio (FCR), egg external quality (eggshell quality, egg shape index, and specific gravity), egg internal quality (egg yolk color, Haugh unit, albumin and yolk index, albumin weight and height, and yolk weight), serum biochemicals (total cholesterol, triglyceride, glucose, albumin, uric acid, and total protein), antioxidant activity (SOD, GSHPx, and malondialdehyde [MDA]), cecum microbial population (*Lactobacillus, Bifidobacteria, E. coli*, and *Salmonella*), and intestinal morphology (villi height and crypt depth).

### Statistical analysis

We analyzed the data using a meta-analysis technique based on the mixed model methodology [35]. Random effects and fixed effects were applied to the experimental groups and the EO levels, production phase, and chicken breed, respectively. Data were analyzed using SAS^®^ On Demand for Academics, and p < 0.05 was considered statistically significant. We applied the root mean square error to the statistical model. When the quadratic regression model results were not significant, a linear regression model was used. We determined the relationship between the variables using the slope and intercept.

Assessment of the results was performed using the following statistical model:

Yijk = μ + si + τj + sτij + β0 + β1Xij + β2X2ij + biXij + βj Xij + eijk

where Yijk = Dependent variable; μ = Mean of data; si = Random effect (experiments group); τj = Fixed effect of the j^th^ level of factor; sτij = Random interaction between the i^th^ experiment and the j^th^ level of factor; β0 = Intercept; β1 = Coefficient of linear regression for fixed effect of Y on X; β2 = Coefficient of quadratic regression for fixed effect of Y on X; Xij = Continuous predictor value of the variable (EOs level); bi = Random effect of experiment I on the coefficient regression of Y on X in experiment I; βj = j^th^ level effect of the discrete factor τ on the regression coefficient of fixed effect; and eijk = Unexplained residual error [35].

## Results

### Production performance and egg quality

**Figure-2:**
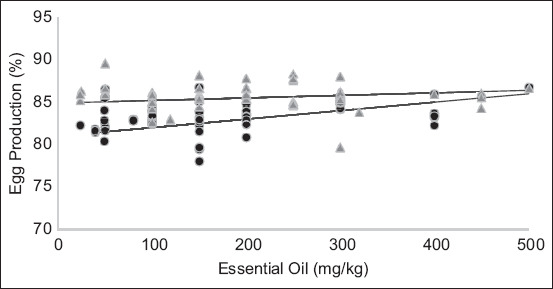
Effects of essential oil (EO) levels and lightweight (▲) or semi-heavyweight (●) laying hens on egg production. Equation of light laying hen breeds: Egg production = 84.94 + 0.003 × EO (mg/kg) (n = 93, p < 0.05). Equation of semi-heavy laying hen breeds: Egg production = 81.04 + 0.01 × EO (mg/kg) (n = 49, p < 0.05).

**Figure-3:**
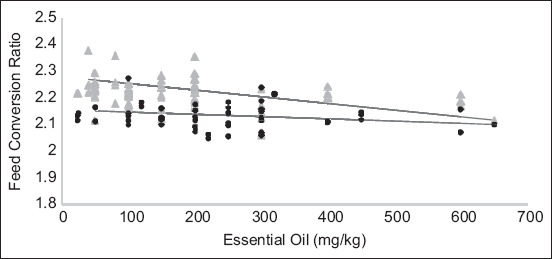
Effects of essential oil (EO) levels and lightweight (●) or semi-heavyweight (▲) laying hens on feed conversion ratio (FCR). Equation of lightweight laying hen breeds: FCR = 2.156−0.000085 × EOs (mg/kg) (n = 53, p < 0.05). Equation of semi-heavy weight laying hens breeds: FCR = 2.28−0.0003 × EOs (mg/kg) (n = 89, p < 0.05).

According to the present meta-analysis, increasing the EO level had a favorable effect on hen-day production, FCR, egg weight, and egg mass (Table-2). Hen-day production and feed intake increased linearly with increasing EO concentration (p = 0.05). Similarly, the FCR value decreased linearly (p < 0.05), demonstrating a favorable effect of EOs. Simply, EOs increased feed efficiency. In addition, egg weight and mass quadratically increased (p < 0.05), which was consistent with an increase in EOs. Furthermore, the effects of EOs on egg production, FCR, and egg mass were associated with the laying hen breed (p < 0.05) (Figures-2 and 3), whereas egg weight was influenced by the interaction between EOs level and laying hen production phase (p < 0.05). In general, there were no adverse effects of EOs on egg quality (Table-3). However, inclusion of EO linearly increased shell thickness, percentage of shell weight, and shell strength (p < 0.05).

**Table-2:** Effect of dietary EOs on laying hen productive performance.

Parameter	n	Intercept	SE intercept	Slope	SE slope	p-value	RMSE	Interaction		Trend	Model

								EOs x phase	EOs x breed		
Egg production (%)	212	82.6	1.04	0.0075	0.0014	<0.001	5.516	0.244	0.012	I	L
Feed intake (g/hen)	212	113	1.19	0.0028	0.0011	0.013	4.923	0.872	0.799	I	L
FCR	212	2.22	0.03	−0.00017	0.000041	<0.001	0.177	0.834	0.045	D	L
Egg mass (g/hen/day)	212	49.6	0.73	0.013 −0.00002	0.00199 0.0000044	<0.001	4.28	0.03	0.002	I	Q
Egg weight (g/egg)	188	58.1	0.62	0.0053 −0.0000063	0.00095 0.0000016	<0.001	2.456	0.039	0.937	I	Q

n=Number of treatments, RMSE=Root mean square error, SE=Standard error, L=Linear, Q=Quadratic, I=Increase, D=Decrease, EOs=Essential oils, FCR=Feed conversion ratio

**Table-3:** Effect of dietary EOs on laying hen egg quality.

Parameter	n	Intercept	SE intercept	Slope	SE slope	p-value	RMSE	Interaction		Trend	Model

								EOs x phase	EOs x breed		
Yolk weight (%)	30	27.7	0.76	−0.00108	0.0012	0.380	3.40	0.537	0.875	D	L
Albumin weight (%)	27	63.3	0.20	−0.00054	0.0003	0.097	0.841	0.691	0.367	D	L
Shell thickness (mm)	106	0.36	0.01	0.000027	0.000008	0.002	0.365	0.365	0.171	I	L
Shell weight (%)	78	9.80	0.24	0.00069	0.00023	0.003	0.630	0.869	0.691	I	L
Yolk color	66	8.17	0.55	0.00018	0.00023	0.449	0.624	0.649	0.462	I	L
Shell strength (kg)	88	4.12	0.18	0.00048	0.00016	0.005	0.471	0.381	0.649	I	L
Yolk index (%)	41	43.5	0.89	0.0011	0.0009	0.229	2.788	0.203	0.792	I	L
Haugh unit	100	80.2	1.09	0.0010	0.0013	0.423	5.010	0.969	0.424	I	L
Egg shape index (%)	47	74.8	0.41	0.00042	0.00093	0.658	2.339	0.843	0.786	I	L
Albumin height (mm)	56	6.82	0.20	0.00013	0.00021	0.529	0.483	0.967	0.189	I	L
Specific gravity (g.cm^-3^)	28	1.08	0.004	0.0000047	0.000018	0.799	0.028	0.795	0.650	I	L

n=Number of treatments, RMSE=Root mean square error, SE=Standard error, L=Linear, I=Increase, D=Decrease, EOs=Essential oils

### Biochemical and oxidative stress parameters

The EOs did not affect triglyceride, albumin, uric acid, or total protein levels (Table-4). However, inclusion of EOs linearly decreased glucose and total serum cholesterol levels (p < 0.05). Similarly, EO caused a linear increase in SOD and GSHPx enzymes (p < 0.05). As a result, MDA levels, an indicator of oxidation products, quadratically decreased (p < 0.05).

**Table-4:** Effect of dietary EOs on laying hen serum biochemicals and antioxidants parameters.

Parameter	n	Intercept	SE intercept	Slope	SE slope	p-value	RMSE	Interaction		Trend	Model

								EOs x phase	EOs x breed		
Total cholesterol (mg/dL)	35	166	26.7	0.188	0.088	0.044	130.09	0.265	0.080	I	L
Triglycerides (mg/dL)	30	897	50.6	−0.0024	0.251	0.992	305.22	0.658	0.479	D	L
Glucose (mg/dL)	29	565	90.2	−0.170	0.080	0.040	111.59	0.153	0.990	D	L
Albumin (mg/dL)	30	3.69	0.83	−0.00079	0.0004	0.077	0.506	0.051	0.354	D	L
Uric acid (mg/dL)	25	6.45	0.48	0.00011	0.0012	0.931	1.409	na	0.679	I	L
Total protein (mg/dL)	27	6.13	1.00	0.0025	0.0019	0.203	2.213	0.887	0.865	I	L
SOD (U/mL)	21	64.6	6.47	0.0117	0.0039	0.009	8.038	0.067	0.576	I	
GSHPx (U/mL)	21	22.9	6.73	0.0043	0.0014	0.009	2.935	0.765	0.229	I	L
MDA (nmol/mL)	21	4.83	0.71	−0.0032 0.000006	0.0013 0.0000022	0.019	1.066	0.060	0.489	D	Q

n=Number of treatments, RMSE=Root mean square error, SE=Standard error, na=Not available, L=Linear, Q=Quadratic, I=Increase, D=Decrease, EOs=Essential oils, SOD=Superoxide dismutase, GSHPx=Glutathione peroxidase, MDA=Malondialdehyde

### Microbial population and intestinal morphology

EO inclusion had a beneficial effect on the cecal microbial population and intestinal morphology (Tables-5 and 6). The population of *Lactobacillus* showed an increasing trend, whereas that of *Salmonella* showed a decreasing trend. Moreover, beneficial effect of EOs on intestinal bacteria was associated with laying hen breed (p < 0.05). In addition, increasing EO levels resulted in a linear improvement in the height-to-crypt depth ratio of duodenal and jejunum villus (p < 0.05). No significant effect was observed on villus height (jejunum and ileum), crypt depth (duodenum, jejunum, and ileum), or the villus height-to-crypt depth ratio (duodenum and ileum).

**Table-5:** Effect of dietary EOs on laying hen cecum microbial population (log CFU/g).

Parameter	n	Intercept	SE intercept	Slope	SE slope	p-value	RMSE	Interaction		Trend	Model

								EOs x phase	EOs x breed		
*Lactobacillus*	20	9.86	1.69	0.0012	0.0010	0.265	1.429	0.265	0.005	I	L
*Bifidobacteria*	20	8.63	0.62	0.0011	0.00095	0.278	1.336	0.278	0.241	I	L
*Escherichia coli*	20	8.05	1.16	−0.00086	0.0012	0.492	1.724	0.160	0.114	D	L
*Salmonella*	20	6.78	1.34	−0.0015	0.0011	0.191	1.535	0.496	0.051	D	L

n=Number of treatments, RMSE=Root mean square error, SE=Standard error, L=Linear, I=Increase, D=Decrease, EOs=Essential oils, CFU=Colony forming unit

**Table-6:** Effect of dietary EOs on laying hen intestinal morphology (μm).

Parameter	n	Intercept	SE intercept	Slope	SE slope	P-value	RMSE	Interaction		Trend	Model

								EOs x phase	EOs x breed		
Duodenum											
Villi height	26	1048.02	89.25	0.416	0.122	0.003	230.72	0.089	0.792	I	L
Crypt depth	26	80.97	12.34	0.0082	0.012	0.511	23.09	0.108	0.394	I	L
VH: CD	26	14.68	1.91	0.00395	0.0024	0.122	4.60	0.345	0.582	I	L
Jejunum											
Villi height	20	756.86	86.74	0.065	0.156	0.682	233.70	0.396	0.397	I	L
Crypt depth	20	74.33	9.62	−0.0220	0.0131	0.119	19.61	0.369	0.667	D	L
VH: CD	20	10.84	1.55	0.00451	0.0019	0.033	2.840	0.361	0.364	I	L
Ileum											
Villi height	20	560.89	56.71	0.059	0.0959	0.545	154.29	0.984	0.984	I	L
Crypt depth	20	92.03	22.52	−0.0062	0.0138	0.658	22.05	0.134	0.134	D	L
VH: CD	20	8.68	2.41	−0.0023	0.0013	0.100	2.12	0.065	0.065	D	L

n=Number of treatments, RMSE=Root mean square error, SE=Standard error, L=Linear, I=Increase, D=Decrease, EOs=Essential oils, VH=Villi height, CD=Crypt depth

## Discussion

### Laying hen productive performance

EOs are used in poultry nutrition due to their antibacterial, anti-inflammatory, antioxidant, and digestive enzyme-stimulating properties. In this study, EO inclusion linearly increased egg production, feed intake, and feed efficiency and quadratically increased egg weight and egg mass. The inclusion of 300 mg/kg thymol and carvacrol EOs increased egg production, feed intake, and feed efficiency [6], whereas the addition of 100 mg/kg EOs [21] and 250 mg/kg thymol [37] increased the productive performance of laying hens. In addition, EO blend containing thymol and carvacrol in the diet significantly improved egg weight and egg mass [9]. Xiao *et al*. [34] reported that the beneficial effects of EOs on egg production, egg mass, and weight were associated with their ability to improve ovarian function and nutrient digestion in the gastrointestinal tract. In addition, the positive impact of EOs on laying hen performance may be associated with the bioactive compounds in EOs that stimulate pancreatic digestive enzyme secretion and improve intestinal morphology [38]. Hashemipour *et al*. [39] reported a significant increase in maltase, amylase, and trypsin activities in broilers treated with commercial EO blends.

In terms of intestinal morphology, our meta-analysis also confirmed that the inclusion of EOs linearly increased villus height, crypt depth, and height-to-crypt depth ratio (Table-6). Strengthening intestinal health and the availability of digestive enzymes improves nutrient digestibility and absorption, thereby satisfying the maintenance and production requirements of laying hens. With regard to feed intake parameters, EOs also improve feed palatability through flavor characteristics that boost feed intake or suppress palatability depending on the dose, age, and chicken breed [11].

The effects of EO inclusion on laying percentage and FCR were associated with laying hen breeds, indicating different requirements for different breeds, as shown in Figures-2 and 3. Bozkurt *et al*. [17] observed a significant interaction between the administration of antibiotic growth promoters and laying hen breed, in which the egg production response was higher in the light breed than in the semi-heavy breed. The reason for this is unclear due to the lack of relevant information. However, this may be due to differences in the physiological responses of different body masses between light and heavy breeds. For example, Gonçalves *et al*. [40] reported that laying hens with light body mass had the advantage of dissipating heat compared with hens with heavy body mass. Under these conditions, light-type chickens may be more sensitive to the presence of EOs, resulting in high egg production, feed efficiency, and egg mass. However, there was an interaction between the EO levels and the laying hen production phase (age), but not with the laying hen breed, which affected egg weight. Hence, this finding suggests that dietary EOs have an increased effect on egg weight based on laying hen age and not laying hen breed. This is also in line with the fact that egg size improves with increasing laying hen age [41].

### Egg quality

The current meta-analysis confirmed that the inclusion of EO linearly improves eggshell weight, thickness, and strength. Improving the quality of eggshells at different chicken or breed ages is critical because the weight of the shell naturally decreases with increasing egg size during the production cycle [42], making the eggshells thin and brittle and crack easily. Shell percentage and thickness significantly increased with 150 mg/kg cinnamon oil diet [32] and 200 mg/kg oregano EOs [26]. This is in line with the findings of the current study, which suggests that the beneficial effect of EOs on intestinal health is favorable for the absorption of minerals, especially calcium, which is the main component of eggshells. EO supplementation increases Ca mineral concentrations in the liver [43] and serum of birds [44]. Olgun and Yildiz [45], further, emphasized that the inclusion of EOs decreases Ca excretion and increases its bioavailability. In addition, EO is an excellent solvent for Vitamin D, calcium absorption [43], and eggshell formation.

### Serum biochemical and oxidative stress parameter

The present meta-analysis demonstrated that the inclusion of EOs significantly increases serum cholesterol and decreases glucose levels. Our results are consistent with the inconsistent findings of the previous studies on the effects of EOs on serum cholesterol levels. Herkel *et al*. [43], Migliorini *et al*. [25], and Ghanem *et al*. [32] reported that serum cholesterol levels in laying hens significantly increased with increasing EO concentrations. In contrast, Ghanima *et al*. [6] reported that rosemary and cinnamon administration significantly reduced serum cholesterol levels compared with the control. The increase in cholesterol levels may be explained by the ineffectiveness of EOs in inhibiting 3-hydroxy-3-methylglutaryl CoA reductase, which limits cholesterol synthesis [46]. According to Migliorini *et al*. [25], an increase in cholesterol triglycerides is caused by a high proportion of very low-density lipoprotein, which is the main triglyceride transporter.

The increase in antioxidant enzymes (SOD and GSHPx) with the application of herbal extracts containing EOs was attributed to the antioxidant potential of phenolic terpenoid compounds such as thymol, carvacrol, menthol, rosmarinic acid, and eugenol [2]. The antioxidant mechanism of EOs is related to their ability to donate hydroxyl groups to peroxy radicals and activate antioxidant enzymes, such as SOD and GSHPx. Phenolic terpenoids act as an external mechanism that acts as the cell’s first defense against free radicals and protect cells from oxidative damage. However, under acute oxidative stress, these external mechanisms cannot effectively deal with excess free radicals (reactive nitrogen and oxygen species). Therefore, an endogenous mechanism for the enzymatic pathway, including the activation of catalase enzymes, GSHPx, and SOD, is urgently needed under these conditions. These internal antioxidant enzymes protect cells against oxidative stress through their free radical-scavenging activities. Reactive oxygen species damage cells by interacting with proteins and lipids, resulting in changes in their molecular structure and activities [47, 48]. The presence of MDA is one of the most important indicators of oxidative damage caused by free radicals, and this meta-analysis demonstrated that EO administration significantly reduces MDA levels.

The antioxidant capacity of EOs may also be associated with one of the factors causing decreased serum glucose levels. Nirupama *et al*. [49] reported that oxidative stress triggers an increase in glucose synthesis or hyperglycemia, which is utilized to fulfill the increasing energy demands under stress conditions. Chand *et al*. [50] observed an increase in serum glucose in chickens exposed to heat stress, which may have been due to an increase in adrenaline, which triggers gluconeogenesis in cells. However, contradictory studies have reported that providing essential savory drinking water had no effect [51] and decreased serum glucose levels in chickens [52].

### Intestinal morphology and microbial population

Intestinal health indicators, such as villus height and crypt depth, which play critical roles in the absorption of nutrients can be detected based on morphology. Our results are consistent with those reported by Arslan *et al*. [7], who showed that a diet of 200 mg/kg EOs improved the height of the duodenum villi and decreased the height of the jejunum villi; however, it had no effect on the height of the ileum villi. Sun *et al*. [53] discovered that administration of 60 mg/kg EOs enhanced the jejunum villus height and the villus height-to-crypt depth ratio. In addition, the high duodenal villi of laying hens were improved by the application of oregano EOs at doses of 100 mg/kg [20] and 400 mg/kg [27]. Increased crypt depth is an indicator of the rate of intestinal cell renewal in response to inflammation and epithelial cell shedding [54]. In addition, an increase in villus height and width was positively correlated with increased nutrient absorption in the intestine due to the expansion of the absorptive surface. The beneficial effects of EOs on intestinal villi can be attributed to their antioxidant and antimicrobial properties, which reduce the toxic effects of microbial pathogens [55, 56]. For example, cinnamaldehyde EOs protect villi from hydrogen donors and promote antioxidant enzyme activity [57]. Peng *et al*. [58] suggested that intestinal morphological alterations, such as villus atrophy and crypt hyperplasia, lead to the invasion of pathogenic bacteria, thereby impairing nutritional digestion and absorption, ultimately inhibiting growth and production.

Under both *in vivo* and *in vitro* conditions, EOs are very effective antimicrobials against Salmonella typhimurium and *E. coli* as well as against the changing intestinal microflora, especially *Lactobacillus* [2, 59]. The antibacterial activities of EOs are related to those of phenolic compounds such as thymol, eugenol, carvacrol, and resveratrol [2]. Phenolic compounds permeate the lipid layer of bacterial cell membranes and alter internal pH and homeostasis. The antimicrobial mechanism of EOs is due to their reaction with protein sulfhydryl groups, which causes cell protein inactivation and microbial growth inhibition, as well as their ability to reduce microbial oxygen consumption, thereby restricting nicotinamide adenine dinucleotide oxidation. In addition, because EOs are hydrophobic, they are partitioned between mitochondria and cell membrane lipids to generate a permeable membrane, leading to leakage of cell contents and cell death [60].

The current meta-analysis confirms that the inclusion of EOs effectively increases egg production, egg weight, egg mass, feed efficiency, and eggshell quality (weight, thickness, and strength). These beneficial effects are related to the ability of EOs to improve intestinal health and antioxidant capacity. Intestinal health was confirmed by an increase in villus height and *Lactobacillus* population, as well as a reduction in *Salmonella* bacteria in the cecum. Increased SOD and GSHPx enzymes and decreased MDA levels in the serum were also confirmed as indicators of antioxidant activity improvement.

## Conclusion

The incorporation of EOs effectively increases egg production, feed efficiency, egg weight, egg mass, eggshell quality, oxidative enzymes, and intestinal health. In addition, the meta-analysis suggests that the proportion of dietary EOs in lightweight laying hens is more effective in improving egg production and feed efficiency compared to semi-heavy laying hens. These results confirm that EOs can be used as natural feed additives for laying hens, potentially replacing antibiotics.

## Authors’ Contributions

AD and EÖ: Conceptualization, methodology, and data curation. AD, EG, and ŞÖ: Writing-original draft. EÖ, AJ, AD, EG, and ŞÖ: Data extraction and writing-review and editing. All authors have read, reviewed, and approved the final manuscript.
